# Digital Health Promotion and Prevention in Settings: Scoping Review

**DOI:** 10.2196/21063

**Published:** 2022-01-28

**Authors:** Anna Lea Stark, Cornelia Geukes, Christoph Dockweiler

**Affiliations:** 1 School of Public Health Centre for ePublic Health Bielefeld University Bielefeld Germany; 2 Faculty V: School of Life Sciences University of Siegen Siegen Germany

**Keywords:** setting approach, health promotion, health prevention, eHealth, internet, behavior change, web-based intervention, technology, mobile phone

## Abstract

**Background:**

Digital technologies are increasingly integrating into people’s daily living environments such as schools, sport clubs, and health care facilities. These settings play a crucial role for health promotion and prevention because they affect the health of their members, as the World Health Organization has declared. Implementing digital health promotion and prevention in settings offers the opportunity to reach specific target groups, lower the costs of implementation, and improve the health of the population. Currently, there is a lack of scientific evidence that reviews the research on digital health promotion and prevention in settings.

**Objective:**

This scoping review aims to provide an overview of research targeting digital health promotion and primary prevention in settings. It assesses the range of scientific literature regarding outcomes such as applied technology, targeted setting, and area of health promotion or prevention, as well as identifies research gaps.

**Methods:**

The scoping review was conducted following the Levac, Colquhoun, and O'Brien framework. We searched scientific databases and gray literature for articles on digital setting–based health promotion and prevention published from 2010 to January 2020. We included empirical and nonempirical publications in English or German and excluded secondary or tertiary prevention and health promotion at the workplace.

**Results:**

From 8888 records, the search resulted in 200 (2.25%) included publications. We identified a huge diversity of literature regarding digital setting–based health promotion and prevention. The variety of technology types extends from computer- and web-based programs to mobile devices (eg, smartphone apps) and telemonitoring devices (sensors). We found analog, digital, and blended settings in which digital health promotion and prevention takes place. The most frequent analog settings were schools (39/200, 19.5%) and neighborhoods or communities (24/200, 12%). Social media apps were also included because in some studies they were defined as a (digital) setting. They accounted for 31.5% (63/200) of the identified settings. The most commonly focused areas of health promotion and prevention were physical activity (81/200, 40.5%), nutrition (45/200, 22.5%), and sexual health (34/200, 17%). Most of the interventions combined several health promotion or prevention methods, including environmental change; providing information, social support, training, or incentives; and monitoring. Finally, we found that the articles mostly reported on behavioral rather than structural health promotion and prevention.

**Conclusions:**

The research field of digital health promotion and prevention in settings is heterogeneous. At the same time, we identified research gaps regarding the absence of valid definitions of relevant terms (eg, digital settings) and the lack of literature on structural health promotion and prevention in settings. Therefore, it remains unclear how digital technologies can contribute to structural (or organizational) changes in settings. More research is needed to successfully implement digital technologies to achieve health promotion and prevention in settings.

## Introduction

### Background

The setting approach is considered a main strategy of health promotion [1]. For its practical implementation, it is important to use an operationalizable and consensual concept of a setting. However, various and sometimes divergent definitions exist [2]. A setting can be defined as a relatively permanent social context of which the members are subjectively aware [3] or as a “...place or social context in which people engage in daily activities in which environmental, organizational and personal factors interact to affect health and wellbeing” [4]. Up to now, there has been a lack of a conceptual and theoretical framework of the setting approach [2]. Health-promoting organizational development therefore often makes use of sociological organizational theories, especially systems theory and structuration theory. Furthermore, organizational change theories are applied, including, for example, the diffusion theory of Everett Rogers or the model of change proposed by Kurt Lewin [5]. Public health theories and models are also used for the implementation of setting-based or complex health-related interventions partly considering structural aspects of the implementation, such as the PRECEDE–PROCEED model [6].

To gain a better understanding of what setting-based health promotion and prevention might look like in practice, Whitelaw et al [7] developed a categorization of 5 different types. The passive model focuses on individuals and their health-relevant behavior. The setting is used as an access to individuals and groups. The active model includes interventions that focus on the health behavior of individuals. However, it assumes that some behavior changes are inhibited or enabled by structural features of the setting. Thus, structural modifications are required. The vehicle model focuses on individual health issues, but interventions and projects are intended to initiate systemic changes. The projects serve as *door openers* for developments at other levels of the setting. The focus is on structural factors and their influence on health, even if individual health issues are addressed first. In the organic model, social systems are understood as the product of the processes of individuals and groups living and acting together. Interventions result in systemic changes that affect the communication culture and community values in the setting. The setting is understood as the framework for health, which can be influenced by setting members. Participation and empowerment of people to affect their environment are key concepts. The comprehensive model assumes that the setting affects health. Settings are understood as superordinate systems in which the individual has hardly any opportunities to affect them himself. Interventions are often implemented from the top down [1,7].

In the last decade, digitization has had a major impact on everyday life in settings and on the research and practice of setting-based health promotion and prevention. Approximately 77% of the population uses the internet on a daily basis, and approximately 90% of the population uses it occasionally [8]. Digital media are the most important source of information, especially among the younger population. With the development of health-related digitization (also known as eHealth), internet-based health information and the use of mobile apps for health topics (also known as mobile health [mHealth]) have made extreme progress and replaced traditional face-to-face sources of information [9-11]. Digitization offers the opportunity to customize health promotion and prevention interventions and adapt them to the needs of individuals, for example, through personalized motivational SMS text messages or reminders. Besides 1-way communication of health information through mass media (eg, videos or e-books), digital health promotion and prevention can be realized with interaction in the form of web-based games or web-based forums. Other eHealth possibilities involve the monitoring of health data, web-based training or classes, digital consultations with health experts, or the provision of health information through (video) games, referred to as serious games [12]. At the same time, settings, for example, organizations and institutions, are facing digital transformation processes concerning structure, management, and organizational development, and new (digital) types of organizations are emerging, for example, virtual organizations [13]. In the health sector, digital transformation affects various organizations such as hospitals (*Hospital 4.0*) and pharmacies (*web-based pharmacies*) [14]. With the increased use of eHealth and mHealth apps, everyday life in general is becoming more and more digitalized, which has various effects on health. An example is the digital gaming environment, where web-based players face new health risks as well as accrue new health benefits [15]. Overall, the digitization and the large number of available digital technologies offer great potential in terms of health promotion and prevention, especially in settings.

However, there is still a lack of a clear and consensual definition for digital technologies in health promotion and prevention on the one hand [16] and a lack of a systematic description of the extent and characteristics of this research field on the other hand. Only a few reviews show the range of this topic and illustrate the urgency of scientific work on this issue [17,18].

As we have stated previously, settings are an important starting point for health promotion and prevention efforts because of their huge impact on people’s health [19] and they can be used for digital health promotion and prevention, but this new research field has not been systematically analyzed yet [20-23]. To effectively use the full potential of digital technologies, it is important to define the framework in which settings can be achieved, changed, and used in the field of digital health promotion and prevention [24]. Therefore, it might be helpful to analyze digital interventions in settings according to existing theoretical frameworks that are known from analog setting-based interventions, for example, the types of setting-based health promotion outlined by Whitelaw et al [7]. There is also an urgent need to identify which (digital) structures and factors in organizations need to be changed to achieve successful digital health promotion [5]. By defining and structuring the field of digital health promotion and prevention in settings, the quality and quantity of digital interventions can improve and increase, respectively, in the future.

### Objectives

The overall objective of this scoping review is to provide an overview of the research targeting digital health promotion and primary prevention in settings. More specifically, it aims to (1) assess the range of scientific literature focusing on digital health promotion and primary prevention in settings regarding various outcomes such as applied technology, targeted setting or living environment (these terms are used synonymously), and area of health promotion and prevention and (2) identify research gaps resulting from these findings.

## Methods

This paper follows the reporting guidelines of the PRISMA-ScR (Preferred Reporting Items for Systematic Reviews and Meta-Analyses extension for Scoping Reviews) checklist [25].

### Scoping Review Methodology

#### Overview

We conducted a scoping review that aims at mapping a broad body of literature, identifying research gaps, and making recommendations for future research [26,27]. We followed the 6-step framework outlined by Levac et al [28], who updated and extended the initial framework developed by Arksey and O’Malley [27]. The steps are described separately in the following sections.

#### Step 1: Identifying the Research Questions

The research questions were developed by 2 reviewers (ALS and CG) through an iterative process that included exploratory literature searches, an exploration of relevant terms and concepts, and thematic discussions. In addition, we interviewed experts in the field of scientific reviews in public health on the thematic content, methodological approach, and research questions.

#### Step 2: Identifying Relevant Studies

The search strategy was developed with the support of a specialized librarian in public health. The following scientific databases were searched:

- MEDLINE (through PubMed)
- CINAHL (through EBSCO)
- SocINDEX (through EBSCO)
- PsycINFO (through EBSCO)
- PSYNDEX (through EBSCO)
- IEEE Xplore
- BASE
- Web of Science

Search terms were identified for the categories *health promotion and prevention*, *living environments and settings*, and *technology and digital*, including Medical Subject Headings terms and other index terms, as well as synonyms. Search terms were combined using the Boolean operators *OR* and *AND*. The search was restricted to articles published in English or German from 2010 to January 2020. To exclude reviews and web-based surveys (that do not report on a technology for health promotion or prevention) the terms *NOT review* and *NOT online survey* were used. Where possible, the database filter *humans* was applied. The exclusion criteria are described in detail in the next section. We also included gray literature. Therefore, we screened websites focusing on the topic of digitization and health promotion or prevention. Websites of governments, health promotion associations, medical or health-related facilities, health insurance companies, and universities and other scientific institutions were screened. The last research step included a screening of the reference lists of all eligible articles. The full search strategy, including the scientific databases and search terms, is provided in Multimedia Appendix 1.

#### Step 3: Study Selection

##### Overview

The references were independently screened by 2 reviewers (ALS and CG) for consistency with predefined inclusion and exclusion criteria, starting with screening the titles and abstracts and followed by screening the full texts. Rayyan (Qatar Computing Research Institute), a web-based application developed to facilitate the review process, was used for this step. Conflicts were discussed between the reviewers (ALS and CG) and with scientific experts according to the inclusion and exclusion criteria. The a priori–defined inclusion and exclusion criteria are described in the next sections using the Population, Concept, and Context principle [29].

##### Concept

The included articles had to report on interventions for health promotion or primary prevention. Primary prevention includes all those interventions that are implemented before the first occurrence of an undesirable condition. Examples are vaccinations for people who do not belong to a risk group or interventions to prevent unhealthy behavior or organizational health risks. Primary prevention must be differentiated from secondary prevention aiming at the early detection or control of diseases and from tertiary prevention targeting the chronification, consequential damage, or relapse of diseases. Methods for disease prevention could be education, normative and regulative proceedings, or economic incentive and sanction mechanisms [30]. All articles concerning secondary or tertiary prevention were excluded. For this reason, we excluded articles focusing on already present risk behaviors such as smoking cessation or weight loss for people who are overweight, as well as articles addressing screenings for early disease detection. Furthermore, a distinction can be made among universal, selective, and indicated prevention. We included universal prevention; for example, if an intervention was implemented in an entire school class with the aim of health promotion or primary prevention. We excluded selective and indicated interventions, which are defined as interventions focusing on people with risk factors or precursors of a disease [30]. We included preventive or health-promoting interventions at specialists’ practices (eg, sexual health clinics) if the intervention was not aimed in particular at people who are already ill or those classified as at risk. Thus, it is possible that healthy people attend a preventive health screening or accompany someone who is ill. Furthermore, it is possible that the health issue that led to their appointment is separate from the primary preventive or health-promoting intervention. Besides primary prevention, articles were included if they reported on health promotion. Health promotion interventions aim at enabling the population to enhance their health by strengthening resources [19]. Interventions focus either on strengthening individuals to be empowered or on augmenting the social, ecological, and economic factors that influence people’s health [31]. In this context, the concept of *healthy settings* has to be mentioned. According to the World Health Organization (WHO), a setting for health is a “place or social context in which people engage in daily activities in which environmental, organizational and personal factors interact to affect health and wellbeing” [4,32]. Rosenbrock [3] follows a broader approach and defines settings as a relatively permanent social context that the members have to be subjectively aware of. This includes rather informal settings such as neighborhoods or groups of people with the same value orientation or life circumstances. In our review, we want to outline the research field in its whole breadth; hence, we will refer to the broader setting definition. This means that we also include more informal settings.

In addition, the included articles had to involve up-to-date digital technologies that are used by the setting members. Digital technologies differ from analog ones in that they “operate on the basis of a discrete code. Usually this code is binary, constructed from sequences of binary digits or bits, each of which can be in one of two states, named 0 and 1” [33]. Therefore, we included web-based programs and mobile apps that are used through a computer, telephone, smartphone, tablet, or other digital devices. Games played on a gaming console were included too. Interventions using videos only were included if these were available through a mobile app (eg, tablet) or were accessible on the web. Interventions using traditional or, rather, obsolete DVDs, television, or radio were excluded.

##### Population

Following the definition of health promotion and primary prevention, articles were included if the intervention targeted healthy people. Therefore, articles focusing on people engaging in risky health behavior or already having symptoms or diseases were excluded.

##### Context

Health promotion or prevention interventions were included if they referred to a setting according to the definitions suggested by the WHO [4] and Rosenbrock [3]. Examples are formal organizations such as schools, universities, and health care facilities. Furthermore, we included less formal settings such as households and neighborhoods because they meet the criteria of a setting by featuring a social context that the setting members (eg, household members or neighbors) are aware of. Articles relating to the workplace as a setting were excluded because the field of health promotion and prevention at work is a separate specific research area within public health. The reviewers had the presupposition that the definitions and characteristics of formal and informal settings might be applicable to digital environments or a digital social context (eg, social media platforms). Therefore, the accordance of the screened articles with the definition of a setting was checked not only for analog conditions, but also for digital conditions. According to the definitions, a criterion is the existence of an accepted social system, namely a given social context that the users of the digital technology are aware of. This criterion was considered fulfilled if the digital technology allowed social interaction among different users of the technology and if the users were aware of the existence of other users. Another criterion was that the social context exists relatively permanently. This criterion was considered fulfilled if the social interaction within the digital technology was not only possible once, but also over a longer period. In accordance with the definition, the identified digital settings could be characterized as formal (such as digital organizations) or informal (such as digital groups of friends).

Besides the Population, Concept, and Context principle, another inclusion criterion concerned the publication type and study design. Nearly all publication types, empirical as well as nonempirical (eg, discussion papers), and study designs were included. The only study design excluded was reviews because the studies included in reviews would have been listed in the relevant databases and would have been identified through our search strategy if they corresponded to our inclusion criteria. In addition, we excluded abstracts and posters because they would not deliver all the required study information. Furthermore, articles had to be published in English or German from 2010 to January 2020. The time restriction was necessary because of the rapid and significant changes that occur in the digital sector.

#### Step 4: Charting the Data

Data extraction was conducted independently by 2 reviewers (ALS and CG). For this purpose, a data extraction form was developed, which was discussed during the expert consultations and updated during the extraction process. The reviewers extracted general publication characteristics, for example, year of publication, country, and publication type or study design on the one hand, as well as specific outcomes that are relevant for the description and mapping of the research field, for example, the type of applied technology, the type of setting, the target group of the intervention, and the area and method of health promotion or prevention on the other hand.

The distinction according to the technology type was made based on the study by O’Neil et al [34], which included mobile devices (eg, smartphones and tablets), computer- and web-based programs, social media apps, and telemonitoring devices. We added gaming consoles to the classification. The method of health promotion and prevention was determined according to the distinction by Leppin [30]. The author first distinguishes whether the behavior or the environment is being changed. Second, the author differentiates among educational interventions (including information provision and training), normative and regulative proceedings (eg, new laws or prohibitions), and economic incentive and sanction mechanisms. To this categorization, Scherenberg [12] adds methods for digital health prevention, including monitoring, reminder, social interaction, and social support (eg, digital challenges or encouragement).

#### Step 5: Collating, Summarizing, and Reporting the Results

Following Levac et al [28], this stage was divided into 3 distinct steps. First, we analyzed the results, including numerical summary analysis. Second, we reported the results focusing on the outcomes that referred to the overall research aims. Third, we considered and discussed the findings and their meaning for future research, practice, and policy. We did not carry out a critical appraisal of the methodological quality of the included articles because the aim of this scoping review is to provide an overview of the research field and not to analyze the effectiveness of interventions.

#### Step 6: Consultation

As mentioned previously, the review process was supported by scientific experts through regular consultations and discussions. The scientific advisory board included a specialized librarian, an expert in conducting literature reviews, and an expert in the field of digital health promotion and prevention.

## Results

### Overview

**Figure 1:**
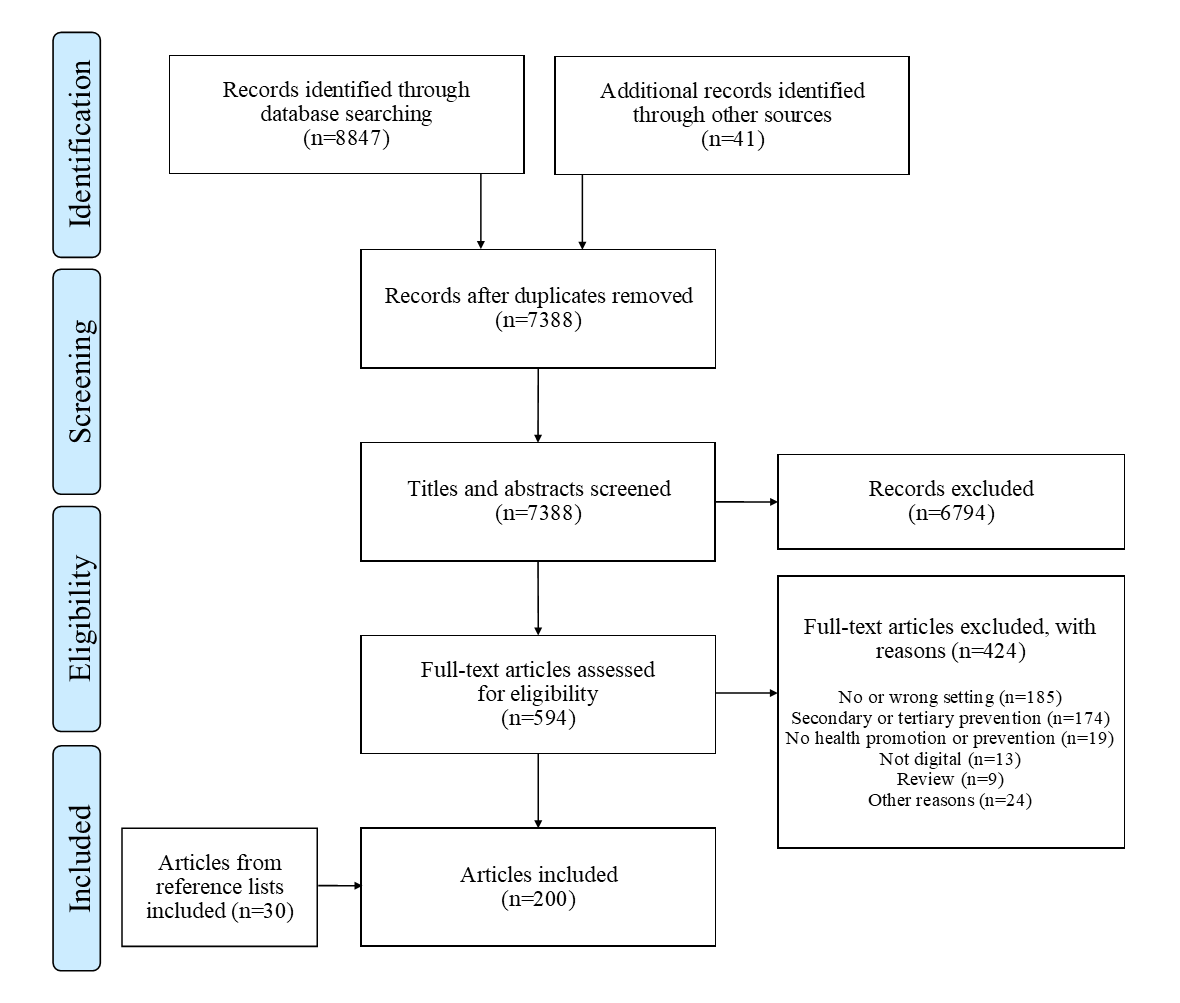
PRISMA (Preferred Reporting Items for Systematic Reviews and Meta-Analyses) flowchart.

The initial database search resulted in the identification of 8847 records, and the gray literature search resulted in the identification of 41 records. From the 8888 articles, 1500 (16.88%) duplicates were removed, leaving 7388 (83.12%) articles eligible for screening by title and abstract. Of these 7388 articles, 6794 (91.96%) were excluded after the screening, leaving 594 (8.04%) articles for the full-text appraisal. Of these 594 articles, after the full-text appraisal, a further 424 (71.4%) were excluded, leaving 170 (28.6%) articles that met the inclusion criteria. In addition, the reference lists of these articles were screened, which led to a further 30 articles being identified; therefore, the literature search yielded 200 articles. The whole review process is summarized in the PRISMA (Preferred Reporting Items for Systematic Reviews and Meta-Analyses) flowchart [35] in Figure 1.

### General Characteristics of the Publications

The articles included in this review were published from 2010 to January 2020. There were 9-28 publications per year in the research area of digital health promotion and prevention in settings (except for 2020 where the search was restricted to January of that year). Whereas the number of publications was 9 and 10 in 2010 and 2011, respectively, it doubled in 2012 and thereafter fluctuated from 16 to 28 publications, with the peak in 2016 and the minimum in 2017. The data extraction chart, including all extracted data used in this review, is provided in Multimedia Appendix 2 [36-235].

In total, we found publications from 25 different countries on 6 continents (based on the country where the first author’s research institution is based). Of the 200 articles, 94 (47%) were published in the United States and 3 (1.5%) in Canada, whereas 96 (48%) were published in Europe. Of these 96 articles, 15 (16%) originated from Germany, 12 (13%) from the Netherlands, 11 (11%) from the United Kingdom, and 6 (6%) from Belgium, whereas other European countries, including Italy, Norway, Denmark, Portugal, Austria, Finland, Ireland, Sweden, Switzerland, Spain, and Greece, each accounted for 5 (5%) or fewer publications. Of the 200 articles, 21 (10.5%) were from Australia, whereas Asian countries, including India, Thailand, Taiwan, Korea, and China, each accounted for 3 (1.5%) or fewer publications; 2 (1%) articles originated from South America (Brazil), and 1 (0.5%) was published in an African country (Uganda).

**Figure 2:**
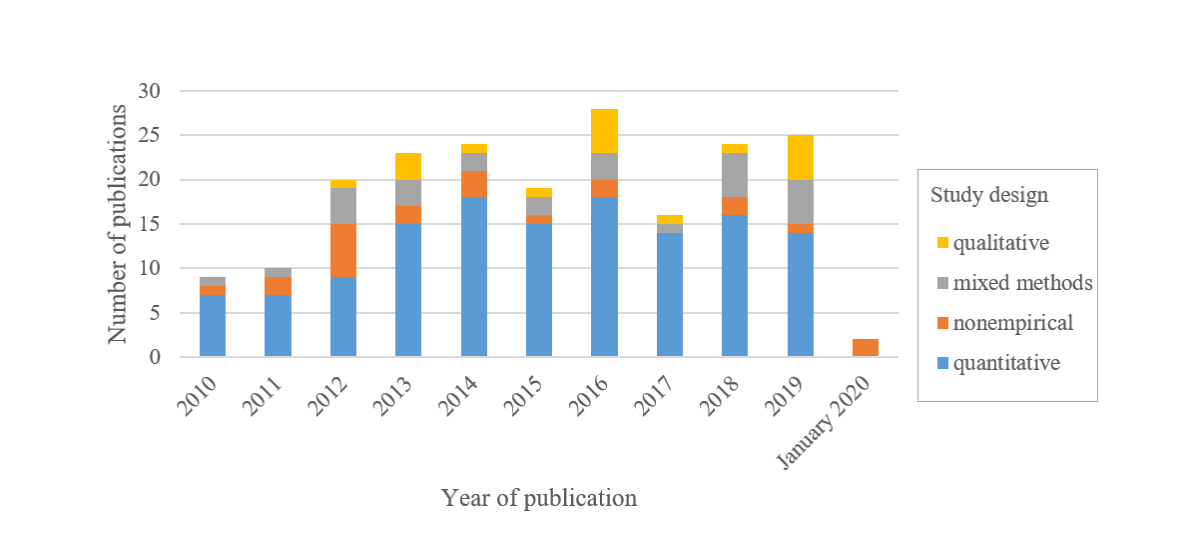
Study designs by publication year (n=200).

Of the 200 articles, 178 (89%) were empirical studies. Most of these 178 studies had a quantitative study design: 65 (36.5%) were randomized controlled trials (RCTs), 22 (12.4%) were cross-sectional studies, 15 (8.4%) were pretest–posttest studies, 13 (7.3%) were secondary data analyses, 11 (6.2%) were study protocols, and 7 (3.9%) were controlled trials. Of the 178 empirical studies, a further 27 (15.2%) followed a mixed methods approach. Of these 27 studies, 4 (15%) were study protocols and 1 (4%) was a secondary data analysis. Of the 178 studies, 18 (10%) had a qualitative design. In addition, among the 200 studies, we found 22 (11%) nonempirical publications, including theoretical or discussion papers, technology or framework descriptions, and thematic overviews. Figure 2 shows the distribution of study designs by publication year.

### Type of Digital Technology Used for Heath Promotion and Prevention in Settings

**Figure 3:**
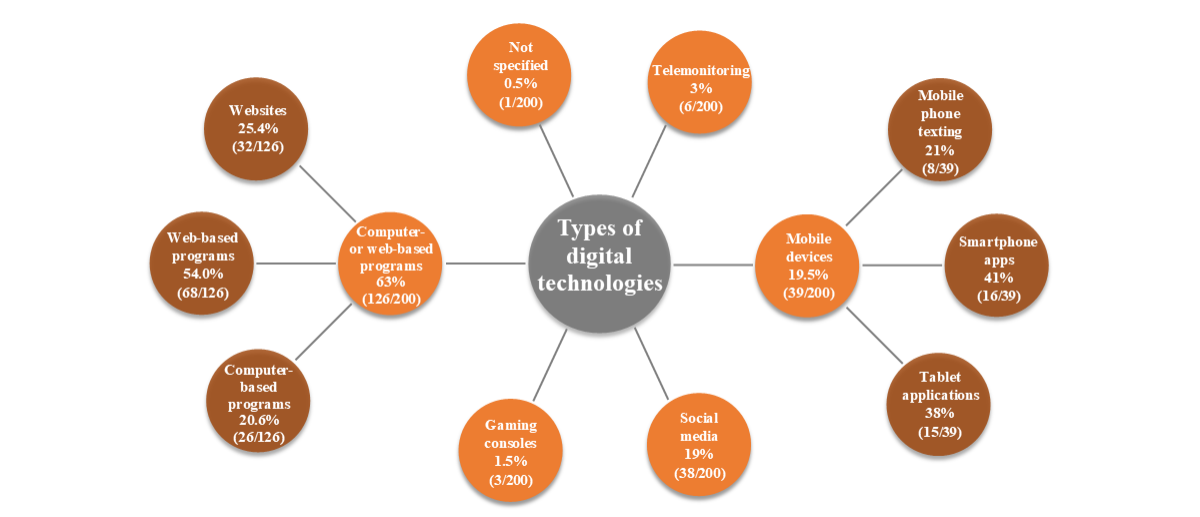
Types of digital technologies (n=213 due to the fact that some publications report on more than one technology type).

As Figure 3 shows, the type of digital technology used for health promotion or prevention can be distributed through mobile devices (eg, smartphones and tablets), computer- and web-based programs, social media apps, telemonitoring devices (eg, external sensors or smartwatches), and gaming consoles. Of the 200 studies, 126 (63%) targeted computer- and web-based programs, 39 (19.5%) a mobile device, and 38 (19%) a social media app, whereas only a small number (6/200, 3%, or fewer) reported on one of the other technology types. As some publications reported on ≥1 technology types, the sum of the percentages adds up to >100% in >200 articles (this also applies to the targeted areas and methods of health promotion and prevention, described in the *Areas and Methods of Digital Health Promotion and Prevention in Settings* section).

Furthermore, these technology types offer different apps that are used for health promotion or prevention purposes. Regarding the technology type *mobile devices*, 41% (16/39) of the publications reported on smartphone apps and 38% (15/39) reported on tablet apps, whereas 21% (8/39) used mobile phone texting. Of the interventions that included a computer- or web-based program, 54.0% (68/126) were web-based programs, 25.4% (32/126) were websites, and 20.6% (26/126) were computer-based programs.

There were different apps and programs with a variety of digital functions and options for users mentioned in the publications, which cannot be described in detail in this review. A few apps are presented as examples here. An example is the use of a smartphone with an integrated geographic information system for the promotion of physical activity. The underlying idea is that specific environmental conditions of a neighborhood can promote physical activity. These features, referred to as neighborhood walkability, can be measured through a geographic information system and used to improve the environmental conditions [36]. Other interventions used computer-based programs, including virtual reality, for health promotion and prevention purposes. As an example, a study combined stationary cycling at independent living facilities for older adults with virtual reality to create a computer-simulated environment (cybercycling). This intervention, also referred to as an exergame (a digital game that requires one to perform physical exercise), aims at improving the cognitive function of older adults [37]. Another digital intervention concerns serious games, which are digital games with educational objectives played on computers or mobile devices. We identified serious games for various health promotion and prevention purposes, for example, a game tackling childhood obesity in schools [38] or a game on neglected tropical diseases developed for use in a community [39]. As an example for telemonitoring devices, a study focused on the real-time monitoring of children’s physical activity in school measured by smartwatches and supervised by classroom teachers, which, over the course of time, enables teacher-regulated strategies for providing more opportunities for physical activity [40]. A gaming console was used, for example, by children in schools, where active video games were played to enhance their physical activity during school breaks [41]. Another intervention at a preschool investigated the use of a social media platform for the delivery of health-related education materials to augment a classroom-based obesity-prevention curriculum [42].

### Target Groups and Targeted Settings in Digital Health Promotion and Prevention

The analysis identified 2 categories of target groups for digital health promotion and prevention interventions. First, there were interventions where the users of the technology were the direct target group for health promotion or prevention. Examples of direct target groups were adolescents, adults, children, church members, community members, expatriates or travelers, men, mothers or pregnant women, older adults, patients, school or university students, vacationers, women, and young adults. In addition, some publications focused on people from a specific cultural, linguistic, or sexual background (eg, refugees, bisexual men, and people living in rural areas). Others had >1 target group or did not specify the target group. Of the 200 included studies, most (48/200, 24%) targeted school students, followed by those targeting older adults (18/200, 9%), university students (17/200, 8.5%), and adults in general (15/200, 7.5%). Second, there were interventions where the technology users were not the direct target group for health promotion or prevention. Instead, the technology users (as multiplicators or mediators) were empowered or supported through the use of the technology to ensure that other people received health promotion or prevention. Examples of multiplicators or mediators were parents, teachers, doctors, and childcare center directors.

The aforementioned target groups already indicate some settings where digital health promotion and prevention interventions take place. The settings were divided into formal and informal settings. The formal analog settings that we identified were schools, universities, and other educational settings; community facilities (eg, churches or prisons); health care facilities (eg, primary care, dental, sexual health, or vaccine clinics); and care for older adults or childcare facilities. Informal analog settings were communities or neighborhoods and the household or home. We identified several digital technologies that might be characterized as a digital setting at the time of publication. These were web-based distance learning universities; virtual reality apps; and social media, including apps, websites, and web-based platforms or web-based programs that offered discussion boards, forums, or other ways of social interaction. The web-based distance learning setting can be characterized as a formal digital setting.

Overall, with 31.5% (63/200) of the publications reporting on it, social media was the most frequent setting in digital health promotion and prevention. Another 19.5% (39/200) of the publications reported on schools as a setting, 12% (24/200) on neighborhoods or communities, and 6.5% (13/200) on health care facilities. Furthermore, 6% (12/200) focused on the informal setting *household or home* and 3% (6/200) on the formal setting *universities*. Other publications thematized interventions that used combinations of analog and digital settings that we refer to as blended settings. For instance, interventions in the community were often complemented with a digital app that itself represented a digital setting (eg, a social media platform). The same combinations were found for schools and universities.

### Areas and Methods of Digital Health Promotion and Prevention in Settings

The included articles dealt with different areas of health promotion and prevention. Physical activity interventions were the most commonly applied, with 40.5% (81/200) of the publications thematizing them. Other often considered areas were nutrition in 22.5% (45/200) of the publications; sexual health in 17% (34/200); substance use, for example, alcohol or drug use, in 10% (20/200); weight in 9.5% (19/200); and mental health in 5.5% (11/200). Only a few publications described interventions for health promotion or well-being in general (9/200, 4.5%), for vaccination (8/200, 4%), and for various risk factors at once (10/200, 5%). Other health topics that were tackled rarely were bone and oral health, sound and sun exposure, cognitive function, fall prevention, sleep, and transmission of diseases (17/200, 8.5%; Multimedia Appendix 3).

Besides the areas of health promotion and prevention, we analyzed the methods used to achieve health promotion and prevention among the target group. Most of the publications reported on interventions that combined several of the following methods: environmental change; providing information, social support, training, or incentives; and monitoring. The provision of information was the most used method (181/200, 90.5%). It aimed at improving the knowledge of the target group regarding a specific health promotion or prevention topic and thereby changing health behavior. Of the 200 interventions, 79 (39.5%) offered some form of social support, for example, through connecting people with the same health problem or the same health goals to motivate the target group. Approximately every fifth intervention (41/200, 20.5%) used monitoring as a method for health promotion and prevention. By giving users the opportunity to collect, visualize, and control health-related data over a longer period, monitoring enabled the user to recognize progress and stay on track. Of the 200 articles, 30 (15%) aimed at changing environmental conditions, for example, a change in the supermarket environment [43] or the school environment [44]. Only a few interventions used incentives as a mechanism to influence health behavior (8/200, 4%) or provide physical or mental training (19/200, 9.5%).

The different methods of health promotion and prevention indicate that one should further distinguish between structural and behavioral health promotion and prevention in settings. Structural health promotion and prevention is realized by changing setting-based or organizational structures. Only a small percentage (30/200, 15%) of the identified articles dealt with structural health prevention. Of these 30 articles, 24 (80%) focused on environmental or organizational changes in analog settings such as neighborhoods, supermarkets, schools, childcare centers, and home environments. An example is a smartphone app that enabled community members to document neighborhood features through geocoded photographs. The intervention aimed at identifying and changing aspects of the physical and social environment that influenced the health behavior of the community members [45]. Only a few articles (6/30, 20%) discussed structural changes in digital settings, for example, social networking sites.

Behavioral health promotion and prevention interventions were used in 85% (170/200) of the included articles. In these behavioral interventions, the setting served as a place to reach a specific target group and offer them an intervention for changing their health behavior. The provision of a tablet in the waiting room of a sexual health clinic that provided access to a website with information for safer sex aiming at a change in sexual behavior is an example [46].

## Discussion

### Principal Findings

Our results provide an initial basis for finding a consensus on the terminology of digital health promotion and prevention in settings through further research. The overall objective of this scoping review is to provide an overview of research targeting digital health promotion and primary prevention in settings. It focuses on assessing the range of scientific literature regarding various outcomes such as applied technology, targeted setting, and area of health promotion or prevention as well as on identifying research gaps that result from these findings. The results provide a first overview of scientific research on digital health promotion and prevention in settings. We have shown the wide range of applications of digital technologies in settings and identified research gaps. We systematically searched for empirical and nonempirical publications from 2010 to January 2020 and identified 200 references that met our inclusion criteria. The data analysis showed that there is a broad range and diversity of literature on digital health promotion and primary prevention in settings regarding various outcomes. Thus, we found publications focusing on a large spectrum of target groups, settings, and technologies as well as areas and methods of health promotion and prevention.

We discovered a wide range of analog, digital, and blended settings where digital health promotion and prevention interventions were applied. Regarding our results, it must be considered that there is a scientific discourse about the definition and application of the concept *settings for health promotion and prevention*. Whereas some researchers support a broader approach, others recommend a narrower definition that only focuses on formal organizations [1]. As we have stated previously, we chose the broader approach.

To our knowledge, this is the first review focusing on digital health promotion and prevention in settings; therefore, we have to consider other studies and sensibly embed and discuss their results compared with our findings. Looking at digital interventions in the field of health promotion and prevention in general, one can see that settings are rarely taken into account. Thus, Norman et al [236] found in their systematic review that eHealth interventions for physical activity and nutrition were sometimes implemented in analog settings such as communities, schools, primary care, or workplaces. In addition, Bailey et al [237] identified in a scoping review that digital interventions for sexual health promotion were delivered in various analog settings, including schools, universities, and health care facilities; through web-based conditions; or through a combination of both, which supported the results of our scoping review.

There is a lack of a (uniform) definition of the terms *digital setting* and *blended setting*; therefore, a way has to be devised to bring together previous knowledge in this area and our results. In line with our presuppositions and results, Loss et al [47] concluded that social networking sites might be categorized as a setting for health promotion and prevention. Other than this publication, the existing scientific literature lacks an extensive and in-depth discussion about the existence and forms of digital (and blended) settings. What can be stated is that the most commonly found digital setting in this review, namely social media, is a widely discussed and used platform for health promotion and prevention interventions in terms of behavioral interventions. It is considered that social media interventions have the potential to facilitate access to health information as well as to expand the reach, improve the efficacy, and lower the costs of health promotion and prevention interventions [238-241]. However, it must be taken into account that the perspective of organizations (besides the workplace) has so far been largely absent from the scientific literature. The digitization of a formal organization in the field of health promotion and prevention is accompanied by massive structural changes. So far, however, these have not been scientifically investigated and evaluated—apart from digital health promotion in the workplace. For future research in the field of digital setting-based health promotion and prevention, this perspective must be taken into account.

What is most striking is the wide range of technologies used to implement health promotion and prevention in settings. These range from simple SMS text messages and smartphone apps to web-based programs offered on tablets in the waiting areas of health care providers. The existence of so many different technologies in the field of health promotion and prevention was identified in other reviews as well [34,242]. This diversity results from the rapid development of technologies in the health sector in the last few years, and it is reflected in the variety of terms that exist to define digitization in the health sector, for example, digital health, eHealth, mHealth, and Health 2.0. [16]. Another reasonable distinction of technology types comes from Lupton [238], who differentiates health promotion and prevention technologies between Web 1.0 and Web 2.0 applications. Web 2.0 refers to a newer generation of web-based applications that enable interaction and that allow users to generate, create, and share information [48]. This distinction can also be applied to the articles we identified.

Another central finding refers to the distinction between structural and behavioral health promotion and prevention in settings. Thus, most (170/200, 85%) of the publications on digital health promotion and prevention in settings can be categorized as behavioral health promotion and prevention in settings. In many cases, this means that the setting was *used* to reach a specific target group aiming at a change in their behavior. Consequently, we found a lack of articles concerning structural health promotion and prevention in settings, specifically a lack of interventions aiming at a change in setting-based and often organizational structures. It remains unclear how structural changes in settings can be achieved using digital interventions. Among the publications that were oriented toward structural health promotion and prevention in settings, only a few discussed structural changes in digital settings compared with changes in analog settings. This highlights that besides the lack of a scientific discussion on the existence and forms of digital settings for health promotion and prevention (which we have already pointed out), there is a research desideratum on how the structures of digital settings can be changed to improve the health of digital technology users.

Following the categorization of setting-based health promotion by Whitelaw et al [7], most of the digital health-promoting interventions can be classified under *passive model* where the setting is used as an access to individuals to try to get them to change their behavior. Interventions mostly miss out on taking into account the specific structures, social contexts, and interaction possibilities of the setting and cannot therefore be assigned to the other models. Nevertheless, we found a few examples in the literature where the digital setting-based intervention includes characteristics of the other intervention types. Table 1 presents examples of identified studies that were assigned to the types described by Whitelaw et al [7]. This overview demonstrates that existing theoretical frameworks from analog interventions can be used to structure digital health-promoting and preventive interventions in settings. Further research should focus on whether new types of setting-based health promotion emerge as a result of digitization.

**Table 1:** Digital interventions and types of setting-based health promotion (types based on Whitelaw et al [7], following Engelmann and Halkow [1]).

Type	Example of digital health promotion or prevention
**Passive model** Individuals and their health-relevant behavior are at the center of interventions Setting is used as an access to individuals and groups to provide them with the health-promoting intervention Health promotion *in* the setting	**Bailey et al [46]** The intervention consisted of a tablet that was displayed in a waiting room showing a website that aims to increase condom use and reduce sexually transmitted infections The setting *health clinic* was used to access individuals to try to get them to change their health behavior Setting or organizational structures were not considered
**Active model** Focuses on the health behavior of individuals Some behavior changes are inhibited or enabled by structural features of the setting Structural modifications are required	**Bourdeaudhuij et al [49]** An internet-based physical activity intervention was implemented in schools The aim of the intervention was to change health behavior A few structural features of the setting were considered, for example, technical equipment or environmental barriers
**Vehicle model** Focuses on individual health issues Projects are intended to initiate systemic changes Projects serve as door openers for developments at other levels of the setting The focus is on structural factors and their influence on health, even if individual health issues are addressed first	**Templeton et al [50]** The aim was to create a web-based sexual health–promotion intervention to encourage young men in prisons to avail of regular sexual health checkups The health intervention was used to achieve overarching goals, for example, to initiate collaborative partnerships to disrupt structures that lead to health inequities, to fulfill the human rights of prisoners, to develop a health-promoting prison
**Organic model** Social systems are the product of the processes of individuals and groups living and acting together Interventions bring systemic changes that affect the communication culture and community values in the setting The setting is understood as the shaping framework for health, which is also shaped by setting members Participation and empowerment of people to shape their environment are key concepts	**Sheats et al [43]** Citizens use an app to collect data about their neighborhood that facilitate or hinder healthy living They use the findings to advocate for change in partnership with local decision-makers and policy makers Includes community-engaged methods to empower citizens This transforms the possibilities of participation and communication in the community
**Comprehensive model** Health is shaped by setting influences Settings are understood as superordinate systems in which the individual has hardly any possibilities to shape them himself Interventions are often implemented from the top down by the setting policy	**Delany et al [51]** Web-based canteen ordering system in a school is altered with the methods of labeling, placement, and prompting to achieve healthier food choices of students Change in the ordering system as a political decision of the school Intervention is implemented from the top down; decision is not up to the students

We identified a wide spectrum of methods that are applied to achieve health promotion or prevention through digital interventions. It is noticeable that the methods of training and monitoring are mostly used for interventions on physical activity, whereas environmental change was mostly applied as a method within interventions on nutrition. A reason for that could be that there exist monitoring devices that are more effective or easier to use in the area of physical activity, for example, pedometers that automatically count steps compared with devices that monitor food intake. In addition, it is possible that environmental changes are easier implemented or more effective in the area of nutrition, for example, changes in the food environment at home compared with changes in physical activity options in settings. Comparing the methods used in the articles included in this review with those identified by Leppin [30] and Scherenberg [12], it is noticeable that normative and regulative proceedings as well as digital reminders were not applied. This could be because the development and introduction of new laws is difficult to realize through digital interventions and because digital reminders (eg, for vaccinations) are a method for individual health promotion rather than for setting-based interventions.

As we did not analyze the effectiveness of digital health promotion and prevention interventions in settings, this needs to be investigated in future research.

We found that most of the publications were empirical and the most used study design was an RCT. Only a small percentage had a qualitative design. Taking a closer look at the study designs, one can see that only a few studies followed a participatory design and that the RCTs usually analyzed the effectiveness of behavior-changing interventions. Therefore, one could conclude that research on digital health promotion and prevention in settings is at an advanced stage because digital apps are tested on their effectiveness rather than being in a previous stage of developing technologies or more fundamental research. However, as we did not find many articles focusing on structural health promotion and prevention in settings, we cannot generalize this interpretation to the whole research field. As we have stated previously, there is a huge research gap concerning structural health promotion and prevention in settings, and research that is more fundamental is needed in this area. A possible explanation for the huge number of RCTs regarding behavioral health promotion and prevention and the lack of RCTs regarding structural health promotion and prevention could be that the effectiveness of changing health behavior (eg, physical activity) is more easily measurable than the effectiveness of changing structures or processes in formal and informal settings.

### Limitations

This scoping review was conducted in compliance with the standards of the methodological approach outlined by Levac et al [28] and in accordance with the reporting guidelines of the PRISMA-ScR checklist [25]. Nevertheless, it includes some limitations that have to be taken into account.

First, the database search was very challenging because it was difficult to find suitable keywords that reflect the concept of a setting so as to be understood in the field of health promotion and prevention. Especially in combination with the term *technology*, the meaning of the word *setting* was not equivalent to our understanding of the concept. In addition, in none of the databases was the term *setting* well indexed. Another limitation derives from the fact that we searched for the terms *health promotion* and *health prevention* in general. We did not expand our search to specific health promotion areas such as physical activity or mental health. Therefore, we might have missed some articles if the terms *health promotion* or *health prevention* were not listed.

Second, our analysis and results are based on our presupposition that the definition of a setting for health promotion and prevention is applicable in the digital context too. Because of a lack of a definition of the term *digital setting*, the inclusion and exclusion criteria are based on the definitions of the term *setting* provided by Rosenbrock [3] and the WHO [4]. This presupposition has to be confirmed in the future to verify our results.

Third, we limited our search to the area of primary prevention and to health promotion and prevention in settings other than the workplace. These restrictions were chosen because of the huge breadth and diversity of the research area. It is possible that the inclusion of articles on secondary and tertiary prevention as well as articles regarding the workplace would have produced different results. In particular, more research has been conducted on digital health promotion in the workplace, and methods and technologies could potentially be applied to settings outside of the workplace.

### Recommendations for Future Research

As we have stated previously, there are major research gaps in the research area of digital health promotion and prevention in settings, and there is a lack of a sufficient consideration of settings in the field of digital health. In future, the setting approach ought to be given greater importance, especially in the research area of digital health and digital health promotion and prevention. This includes developing definitions for the terms *digital setting* and *blended setting* and specifying their fields of application and the different forms that exist. In addition, key features of digital and blended settings have to be explored, especially with regard to their health impact. We currently know far too little about what elements define a digital setting. We need to further investigate the relevance of social interaction for the definition of digital settings.

Especially, there is a lack of research on structural health promotion and prevention in settings. As described previously, more research is needed on how digital technologies can change structures of formal and informal settings and how structures of digital settings themselves can be changed. Future research should focus on how structural change can be applied (eg, through organizational development), how it can be measured, and what stakeholders (eg, leaders or members of an organization) should be involved in this process. Therefore, a more detailed analysis of the identified articles on structural health promotion and prevention in settings seems useful.

It would also be desirable for future research to consider the development of digital health-promoting tools that focus on all target groups in a setting and that consequently consider the organization as a holistic setting with the different needs and requirements of the various target groups and thus do justice to the complexity and multidimensionality of the setting.

In our scoping review, we successfully mapped the research field of digital health promotion and prevention in settings regarding various outcomes. What remains unclear is the effectiveness of these interventions. Future scientific research should also examine the effects of digital interventions, especially the long-term effects. Digital health promotion and prevention technology can only establish itself when the benefits and risks for a population are proven. In addition, more participatory research is needed to ensure that the needs, wishes, (digital) competences, and possible fears of users are considered in the development, testing, and implementation of these technologies. It would also be interesting to see to what extent participatory research can take place in digital settings or to what extent the WHO’s healthy setting principles (including community participation, partnership, empowerment, and equity [243]) can be achieved through digital tools. Such research would contribute to the acceptance and eventually to the extensive use of digital technologies for health promotion and prevention.

### Conclusions

Our review provides an overview of the research on digital health promotion and prevention in settings. We demonstrated that the research field is complex, heterogeneous, and broad, and our findings reveal research gaps that urgently need to be addressed. A gap concerns study on structural health promotion and prevention in settings, and another concerns the definition of relevant terms, for example, *digital settings*. As the topic of digitization in setting-based health promotion and prevention is multidisciplinary, covering disciplines such as public health, organizational development, and the technology industry, in our opinion, the most important aspect is that different disciplines combine their expertise and work collaboratively to bridge these research gaps. In this context, the participation of potential technology users is unavoidable to guarantee that the users’ needs, especially the needs of the vulnerable, are considered. In addition, it is urgently necessary to clarify which significant improvements will result from the application of digital technologies. So far, there is insufficient basic research that provides information about which technologies in which health areas are best suited to certain settings or target groups. Therefore, in future studies, the great potential of digitization should be further explored based on validated scientific research. Especially, the benefits for the populations’ health and the possibility of making health promotion and prevention interventions more effective and less expensive need to be analyzed.

## Appendix

Multimedia Appendix 1 Search strategy.

Multimedia Appendix 2 Data extraction chart.

Multimedia Appendix 3 Areas of health promotion and prevention (n=254 due to the fact that some publications report on more than one area).

## Abbreviations

mHealth: mobile health
PRISMA: Preferred Reporting Items for Systematic Reviews and Meta-Analyses
PRISMA-ScR: Preferred Reporting Items for Systematic Reviews and Meta-Analyses extension for Scoping Reviews
RCT: randomized controlled trial
WHO: World Health Organization
