# Supplementary material for: Digital Health Promotion and Prevention in Settings: Scoping Review
Source: J Med Internet Res. 2022 Jan 28;24(1):e21063. doi: 10.2196/21063 (PMC8838600; doi:10.2196/21063)
Supplement: Multimedia Appendix 1 [file jmir_v24i1e21063_app1.pdf]

Multimedia Appendix 1: Search strategy

Database	Keywords
Medline	(Mobile Applications [MH] OR Smartphone [MH] OR Cell Phone [MH] OR Internet [MH] OR Telemedicine [MH] OR Computer [MH] OR Information Technology [MH] OR Social Media [MH] OR Virtual Reality [MH] OR digital [TIAB] OR blended [TIAB] OR Internet* [TIAB] OR Online* [TIAB] OR Web-based [TIAB] OR Technology [TIAB] OR technologies [TIAB] OR technology-based [TIAB] OR e-health [TIAB] OR m-health [TIAB] OR Computer* [TIAB] OR „information and communication technology“ [TIAB] OR „information and communication technologies” [TIAB] OR Virtual [TIAB] OR Telemonitoring [TIAB] OR elearning [TIAB]) AND (Primary Prevention [MH] OR Health Promotion [MH] OR Health Education [MH] OR Prevention [TIAB] OR prevent* [TIAB] OR „Promote health“ [TIAB] OR „behavior change“ [TIAB]) AND (Environment [MH] OR Social Environment [MH] OR Setting [TIAB] OR settings [TIAB] OR setting-related [TIAB])
Web of Science	((“Mobile Applications” OR Smartphone OR “Cell Phone” OR Internet OR Telemedicine OR Computer OR “Information Technology” OR “Social Media” OR “Virtual Reality” OR digital OR blended OR Internet* OR Online OR Web-based OR Technology OR technologies OR technology-based OR e-health OR ehealth OR mhealth OR m-health OR “mobile health” OR telehealth OR Computer OR „information and communication technology“ OR „information and communication technologies” OR Virtual OR Telemonitoring OR elearning) AND (“Primary Prevention” OR “Health Promotion” OR “Health Education” OR „Promote health“ OR „behavior change“) AND (Environment OR “Social Environment” OR Setting OR settings OR setting-related) NOT “online survey” NOT Titel: (review))
Cumulative Index to Nursing & Allied Health Literature	(MH Mobile Applications) OR (MH Smartphone) OR (MH Cellular Phone) OR (MH Internet) OR (MH World Wide Web) OR (MH Technology) OR (MH Telemedicine) OR (MH Telehealth) OR (MH Computers and Computerization) OR (MH Information Technology) OR (MH Social Media) OR (MH Virtual Reality) OR (TI digital) OR (TI blended) OR (TI Internet*) OR (TI Online*) OR (TI Web-based) OR (TI technologies) OR (TI technology-based) OR (TI e-health) OR (TI “electronic health”) OR (TI m-health) OR (TI Computer*) OR (TI “information and communication technology”) OR (TI “information and communication technologies”) OR (TI Virtual) OR (TI Telemonitoring) OR (TI elearning) OR (AB digital) OR (AB blended) OR (AB Internet*) OR (AB Online*) OR (AB Web-based) OR (AB technologies) OR (AB technology-based) OR (AB e-health) OR (AB “electronic health”) OR (AB m-health) OR (AB Computer*) OR (AB ”information and communication technology”) OR (AB “information and communication technologies”) (AB Video) OR (AB Virtual) OR (AB Telemonitoring) OR (AB elearning) AND (MH Health Promotion) OR (MH Preventive Health Care) OR (MH Health Education) OR (TI Prevention) OR (TI prevent*) OR (TI „Promote health“) OR (TI „behavior change“) OR (AB Prevention) OR (AB prevent*) OR (AB „Promote health“) OR (AB „behavior change“) AND (MH Environment) OR (MH Social Environment) OR (TI Setting) OR (TI settings) OR (TI setting-related) OR (AB Setting) OR (AB settings) OR (AB setting-related)

IEEE Explore	((("Mesh_Terms": “Mobile Applications” OR Smartphone OR “Cell Phone” OR Internet OR Telemedicine OR Computer OR “Information Technology” OR “Social Media” OR “Virtual Reality”) OR ("Abstract": digital OR blended OR Internet* OR Online* OR Web-based OR Technology OR technologies OR technology-based OR e-health OR m-health OR Computer* OR „information and communication technology“ OR „information and communication technologies” OR Virtual OR Telemonitoring OR elearning)) AND (("Mesh_Terms": Primary Prevention OR Health Promotion) OR ("Abstract": Prevention OR prevent* OR „Promote health“ OR „behavior change“)) AND (("Mesh_Terms": Environment OR Social Environment) OR ("Abstract": Setting OR settings OR setting-related)) NOT "All Metadata": "online survey" NOT "Publication Title": review)
SocINDEX	(SU SOCIAL networking mobile apps) OR (SU Cell Phones) OR (SU Internet) OR (SU World Wide Web) OR (SU Technology) OR (SU Computers) OR (SU Information Technology) OR (SU Social Media) OR (SU Virtual Reality) OR (TI digital) OR (TI blended) OR (TI Smartphone) OR (TI Internet*) OR (TI Online*) OR (TI Web-based) OR (TI technologies) OR (TI technology-based) OR (TI Telemedicine) OR (TI ehealth) OR (TI e-health) OR (TI „electronic health“) OR (TI “Mobile health”) OR (TI mhealth) OR (TI m-health) OR (TI Telehealth) OR (TI Computer*) OR (TI „information and communication technology“) OR (TI „information and communication technologies”) OR (TI Virtual) OR (TI Telemonitoring) OR (TI elearning) OR (AB digital) OR (AB blended) OR (AB Smartphone) OR (AB Internet*) OR (AB Online*) OR (AB Web-based) OR (AB technologies) OR (AB technology-based) OR (AB Telemedicine) OR (AB ehealth) OR (AB e-health) OR (AB „electronic health“) OR (AB “Mobile health”) OR (AB mhealth) OR (AB m-health) OR (AB Telehealth) OR (AB Computer*) OR (AB „information and communication technology“) OR (AB „information and communication technologies”) OR (AB Virtual) OR (AB Telemonitoring) OR (AB elearning) AND (SU Health Promotion) OR (TI Prevention) OR (TI prevent*) OR (TI „Promote health“) OR (TI „behavior change“) OR (AB Prevention) OR (AB prevent*) OR (AB „Promote health“) OR (AB „behavior change“) AND (TI Environment) OR (TI Setting) OR (TI settings) OR (TI setting-related) OR (AB Environment) OR (AB Setting) OR (AB settings) OR (AB setting-related)
PsycINFO	(DE Telemedicine) OR (DE Mobile Health) OR (DE Internet) OR (DE Mobile Applications) OR (De Smartphones) OR (DE Virtual Reality) OR (DE Social Media) OR (DE Computer Games) OR (DE Mobile Phones) OR (DE Computer) OR (DE Technology) OR (TI digital) OR (TI blended) OR (TI mhealth) OR (TI e-health) OR (TI “electronic health”) OR (TI m-health) OR (TI Internet*) OR (TI socialmedia) OR (TI “Mobile phones”) OR (TI Computer*) OR (AB digital) OR (AB blended) OR (AB mhealth) OR (AB e-health) OR (AB “electronic health”) OR (AB m-health) OR (AB Internet*) OR (AB socialmedia) OR (AB “Mobile phones”) OR (AB Computer*) AND (DE Preventive) OR (DE Health Promotion) (DE Health Promotion) OR (TI “behaviour change”) OR (AB “behaviour change”) AND (DE Environment) OR (DE Social Environment) OR (TI Setting) OR (AB Setting)

Psyndex	(DE Telemedicine) OR (DE Mobile Health) OR (DE Internet) OR (DE Mobile Applications) OR (De Smartphones) OR (DE Virtual Reality) OR (DE Social Media) OR (DE Computer Games) OR (DE Mobile Phones) OR (DE Computer) OR (DE Technology) OR (TI digital) OR (TI blended) OR (TI mhealth) OR (TI e-health) OR (TI "electronic health") OR (TI m-health) OR (TI Internet*) OR (TI socialmedia) OR (TI Computer*) OR (AB digital) OR (AB blended) OR (AB mhealth) OR (AB e-health) OR (AB "electronic health") OR (AB m-health) OR (AB Internet*) OR (AB socialmedia) OR (AB Computer*) AND (DE Preventive) OR (DE Health Promotion) OR (DE Gesundheit) OR (DE Gesundheits-Screening) OR (DE Gesundheitsdienste) OR (DE Gesundheitseinstellungen) OR (DE Gesundheitsförderung) OR (DE Gesundheitsverhalten) OR (DE Prävention) OR (TI "behaviour change") OR (AB "behaviour change") AND (DE Environment) OR (DE Social Environment) OR (TI Setting) OR (AB Setting)
BASE	("Mobile Applications" OR Smartphone OR Internet OR Telemedicine OR Computer OR "Information Technology" OR "Social Media" OR "Virtual Reality" OR digital OR blended OR Internet* OR Online OR Technology OR e-health OR m-health OR telehealth OR Telemonitoring OR elearning) AND ("Primary Prevention" OR "Health Promotion" OR „Promote health" OR „behavior change") AND (Environment OR "Social Environment" OR Setting OR settings OR setting-related) doctype:(13 14 17 183)
