# Supplementary material for: Digital Health Promotion and Prevention in Settings: Scoping Review
Source: J Med Internet Res. 2022 Jan 28;24(1):e21063. doi: 10.2196/21063 (PMC8838600; doi:10.2196/21063)
Supplement: Multimedia Appendix 2 [file jmir_v24i1e21063_app2.pdf]

Multimedia Appendix 2: Data extraction chart

No.	Quotation No.	First Author	Year of Publication	Country	Publication Type / Study Design	Setting	Target Group	Topic of Health Promotion / Prevention	Type of Technology	Method of Health Promotion / Prevention
1	36	Duncan	2013	USA	discussion paper	neighborhood	no specific target group	physical activity	online program (with GIS)	environmental change, providing information
2	37	Anderson-Hanley	2012	USA	RCT	independent living facilities, virtual reality	older adults (55+)	cognitive function	online program (game with virtual reality, Cybercycling)	environmental change, training
3	38	Dias	2016	Brazil	cross-sectional	school	students	nutrition, physical activity	computer program (game)	providing information
4	39	Luz	2016	UK	qualitative	community	young people, children	transmission of tropical diseases	computer program (game)	environmental change, providing information, social support
5	40	Byun	2018	USA	RCT (posttest-only)	child care centers	teachers of preschool children	physical activity	tablet application, smartwatch (fitbit)	monitoring
6	41	Duncan	2011	USA	cross-sectional	neighborhood	no specific target group	physical activity	online program (with GIS)	environmental information, providing information
7	42	Swindle	2018	USA	mixed methods	educational facility (Head Start Centers), social media (facebook)	parents of children (preschool)	nutrition	social media (facebook)	providing information, social support
8	43	Sheats	2017	USA	pretest-posttest	neighborhood	older adults	nutrition	tablet application (with GPS)	environmental change
9	44	Yang	2017	Korea	CT	school	students (9-13)	nutrition, physical activity, weight	videos (via Internet)	environmental change, providing information
10	45	King	2016	USA	framework description	community	community members	physical activity	smartphone (app with GPS)	environmental change
11	46	Bailey	2017	UK	qualitative	sexual health clinics (waiting room)	men	sexual health	website (on tablet)	providing information
12	47	Loss	2014	Germany	discussion paper	social media (social networking sites)	no specific target group	health promotion in general	social media (social networking sites)	environmental change, providing information, social support
13	48	Kolt	2013	Australia	study protocol (RCT)	social media (website with social networking)	adults	physical activity	website (with social networking)	environmental information, monitoring, providing information, social support
14	49	Bourdeaudhuij	2010	Belgium	RCT	school	students (12-17)	physical activity	online program	environmental information, providing information
15	50	Templeton	2019	UK	qualitative	prison	men	sexual health	videos (web based animation)	providing information
16	51	Delany	2017	Australia	RCT	school, online cantine	students (5-12)	nutrition	online program (online canteen)	environmental change, providing information
17	52	Allison	2012	USA	discussion paper, conference summary	social media (social networking sites)	adolescents, young adults	sexual health	social media (social networking sites)	providing information, social support
18	53	Apolinário-Hagen	2018	Germany	cross-sectional	distance-learning university	students, university (25-39)	mental health (stress)	online programs (ehealth, mHealth)	providing information, training
19	54	Atkinson	2011	UK	cross-sectional	social media (online TV, Youtube, blogs)	young adults (18-24)	substance use (alcohol)	social media (online TV, Youtube, blogs)	providing information
20	55	Azevedo	2019	Portugal	CT	kindergarten, social media (platform with social networking)	parents with children (3-5)	nutrition	online program (game with social networking)	providing information, training
21	56	Backonja	2014	USA	discussion paper	community	older adults	health promotion in general	no specific technology	environmental change
22	57	Bailey	2016	UK	secondary data analysis (RCT)	sexual health clinics (waiting room)	men	sexual health	website (on tablet)	providing information
23	58	Bamidis	2011	Greece	technology description	home	older adults	cognitive function, physical activity	computer program (game with sensors, ambient assisted living)	monitoring, training
24	59	Baranowski	2016	USA	thematic overview	virtual reality (computer game with social networking)	children	various risk factors	computer program (game with virtual reality, social networking)	monitoring, providing information, social support, training
25	60	Beck	2019	USA	RCT	community	adults	physical activity	mobile phone (text messages)	providing information
26	61	Betsch	2012	Germany	discussion paper, conference summary	social media (discussion boards, web blogs, Facebook, Twitter, Wikipedia, LinkedIn and YouTube)	parents	vaccination	social media (e.g. Linkedin, Facebook, Twitter, YouTube)	providing information, social support
27	62	Bickmore	2013	USA	RCT	ambulatory care practices	older adults (65+)	physical activity	tablet application, kiosk computer	providing information
28	63	Brüll	2016	Germany	qualitative	virtual game	students (18-22)	sexual health	online program (game, virtual world)	providing information
29	64	Buendia Eisman	2013	Spain	RCT (posttest-only)	school	students (12-16)	sun exposure	website	providing information
30	65	Bull	2012	USA	RCT	social media (facebook)	adolescents (16-25)	sexual health	social media (facebook)	providing information
31	66	Buller	2017	USA	RCT (independent/seperate samples)	destination resorts	Vacationers	sun exposure	social media, videos	providing information
32	67	Buman	2013	USA	cross-sectional	neighborhood	older adults (65+)	physical activity	tablet application (with GPS)	environmental change
33	68	Byrd-Bredbenner	2017	USA	study protocol (RCT)	household	parents of children (2-5)	nutrition, physical activity, sleep, weight	website (plus eMail, SMS)	environmental change, monitoring, providing information
34	69	Byrd-Bredbenner	2017	USA	secondary data analysis (RCT)	household	parents of children (2-5)	nutrition, physical activity, sleep, weight	website	environmental change, providing information
35	70	Byrd-Bredbenner	2018	USA	RCT	household	parents of children (2-5)	nutrition, physical activity, sleep, weight	website (plus eMail, SMS)	environmental change, monitoring, providing information
36	71	Calear	2013	Australia	study protocol (RCT)	school	students (14-18)	mental health (anxiety)	online program	providing information, training
37	72	Calear	2016	Australia	RCT	school	students (12-18)	mental health (anxiety, depression)	online program	providing information, training
38	73	Caperchione	2014	Canada	study protocol (RCT)	social media (website with social networking)	adults	physical activity	website (with social networking)	monitoring, providing information, social support
39	74	Carah	2017	Australia	cross-sectional	social media (blog)	no specific target group	substance use (alcohol)	website (with social networking, blog)	providing information, social support
40	75	Carlfjord	2012	Sweden	qualitative	primary health care units	patients	physical activity, substance use (alcohol)	computer program	providing information
41	76	Carr	2013	USA	mixed methods	community	adults (18-65)	physical activity	online program (with geographical mapping)	providing information
42	77	Carr	2010	USA	cross-sectional	neighborhood	no specific target group	physical activity	online program (with GIS)	environmental information
43	78	Cavallo	2012	USA	RCT	social media (facebook)	students, female (<25)	physical activity	social media (facebook)	monitoring, providing information, social support
44	79	Cavallo	2014	USA	secondary data analysis (RCT)	social media (facebook)	students, female (<25)	physical activity	social media (facebook)	monitoring, providing information, social support
45	80	Champion	2016	Australia	RCT	school	students (14-15)	substance use (drugs)	online program	providing information
46	81	Champion	2018	Australia	RCT	school	students (14-15)	substance use (drugs)	online program	providing information
47	82	Champion	2016	Australia	RCT	school	students (13-14)	substance use (drugs)	online program	providing information
48	83	Chandra	2014	India	qualitative	community (slum)	students (16-18)	mental health	mobile phone (text messages or calls)	providing information
49	84	Chen	2014	China	study protocol (RCT)	community (villages)	village doctors	vaccination	smartphone (app)	monitoring, providing information
50	85	Chen	2015	Italy	pretest-posttest	vaccine clinics	parents of children (<18)	vaccination	tablet application	providing information
51	86	Cook	2014	Greece	RCT	school	students (13-18)	physical activity	online program	providing information
52	87	Cook	2014	Greece	RCT	school	students (12-17)	physical activity	online program	environmental change, providing information
53	88	Crawford	2018	Australia	cross-sectional	social media (forum)	expatriates and travelers	sexual health	website (with social networking, forum)	providing information
54	89	Cullen	2017	USA	RCT	household	parents with children (8-12)	nutrition	online program	providing information

55	90	DAK-Gesundheit	2020	Germany	intervention description (on website)	school, social media (website with social networking, blog)	students (15-19)	nutrition, physical activity	website (with social networking, blog)	providing information, social support
56	91	Daley	2018	USA	RCT	social media (online platform with social networking)	pregnant women	vaccination	website (online platform with social networking)	providing information
57	92	Davies	2012	Australia	pretest-posttest	social media (website with social networking)	adults	physical activity	website (with social networking, pedometer)	monitoring, providing information, social support
58	93	Degroote	2018	Belgium	CT	general practice (waiting room)	patients	nutrition, physical activity	tablet application	provididing information
59	94	Dixon	2019	USA	RCT	pediatric clinics	parents of adolescents (11-17)	vaccination	tablet application (videos)	providing information
60	95	Dour	2013	USA	secondary data analysis (RCT)	university	students, university	nutrition, physical activity, weight	online program	providing information
61	96	Duncan	2010	USA	RCT	school	students (10-11)	physical activity	gaming console (Wii)	training
62	97	Dway	2016	Thailand	pretest-posttest	community (low-resource village)	mothers of children (<7)	vaccination	tablet application (cartoons, edutainment)	providing information
63	98	Ehlers	2015	USA	RCT	social media (app with a blog, discussion board, videoconference)	women (30-64)	physical activity	tablet application (with social networking)	providing information, social support
64	99	Ekberg	2013	Sweden	qualitative	social media (online health-promoting community)	adolescents	nutrition, physical activity, weight	social media (online health-promoting community)	providing information, social support
65	100	Ekram	2018	USA	cross-sectional	social media (youtube)	no specific target group	vaccination	social media (youtube)	providing information
66	101	Eschenbeck	2019	Germany	study protocol (RCT)	school	students (>16)	nutrition, physical activity, weight	online program	providing information
67	102	Ezendam	2012	Netherlands	RCT	school	students (12-13)	nutrition, weight	computer program	environmental change, providing information
68	103	Ezendam	2014	Netherlands	secondary data analysis (RCT)	school	students (12-13)	nutrition, weight	computer program	environmental change, providing information
69	104	Fiellin	2016	USA	RCT	virtual game	adolescents (11-14)	sexual health	tablet application (virtual video game)	providing information
70	105	Gabarron	2012	Norway	study protocol (mixed methods)	community, virtual clinic, social media	adolescents	sexual health	virtual clinic (website with geolocation), social media	providing information
71	106	Ganz	2018	Germany	RCT	university	students, university	substance use (alcohol)	online program	providing information
72	107	Garcia-Camacha	2019	Spain	pretest-posttest	community	older adults (65+)	various risk factors	online program	empowerment, providing information, social support, training
73	108	Gilliand	2015	Canada	mixed methods	community	community members	nutrition	smartphone (app)	environmental information, incentives, providing information
74	109	Gold	2012	Australia	cross-sectional	social media (facebook, youtube, twitter, flickr)	young people (16-29), men who have sex with men	sexual health	social media (facebook, flickr, twitter, youtube)	providing information
75	110	Gonzalez	2013	Spain	cross-sectional	social media (online platform with social networking)	patients	nutrition, physical activity	online program (telemedicine + social media components)	monitoring, providing information, training
76	111	Grabowski	2013	Denmark	qualitative	school	students (12-15)	nutrition, physical activity, substance use (alcohol, tobacco)	computer program (game)	providing information
77	112	Graham	2014	USA	mixed methods	social media (website with social networking)	pregnant women	nutrition, physical activity, weight (excessive gestational weight gain)	website (with social networking , blogs)	environmental information, monitoring, providing information, social support
78	113	Graham	2017	USA	secondary data analysis (RCT)	social media (website with social networking)	pregnant women	nutrition, physical activity, weight (excessive gestational weight gain)	website (with social networking , blogs)	monitoring, providing information, social support
79	114	Greaney	2014	USA	secondary data analysis (RCT)	social media (website with social networking)	adults	nutrition, physical activity, substance use (tobacco)	website (with social networking)	providing information, social support
80	115	Grim	2011	USA	CT	university	students, university	physical activity	online program	providing information, social support
81	116	Grindell	2019	UK	qualitative	social media (website with social networking)	older adults, children	physical activity	website (with social networking), pedometer	providing information
82	117	Gubrium	2016	USA	mixed methods	community	adolescents, female (15-21)	sexual health	videos (digital storytelling)	empowerment, social support
83	118	Gustafson	2019	USA	RCT	community (rural), home	students (14-16)	nutrition	mobile phone (text messages)	environmental change, providing information
84	119	Haruna	2019	China	RCT	school	students (11-15)	sexual health	computer program (game)	providing information
85	120	Haruna	2019	China	mixed methods	school	students (11-15)	sexual health	computer program (game)	providing information
86	121	Haug	2018	Switzerland	RCT	school	students (14-16)	substance use (alcohol, tobacco, marijuana)	mobile phone (text messages)	incentives, providing information
87	122	Haug	2017	Switzerland	pretest-posttest	vocational school	students, vocational school	substance use (alcohol, tobacco, marijuana)	mobile phone (text messages)	incentives, providing information
88	123	Helander	2014	Finland	cross-sectional	social media (app with social networking)	no specific target group	nutrition	smartphone (app with social networking)	providing information, social support
89	124	Horstmann	2018	Germany	qualitative	social media (online program with social networking)	no specific target group	wellbeing in general	online program (game with social networking)	monitoring, social support
90	125	Huang	2019	Taiwan	RCT	school	students (11-12)	physical activity	online program (with a geographical information system)	providing information, training
91	126	Hyden	2011	USA	thematic overview	social media (e.g. Facebook, Youtube)	adolescents, young adults	sexual health	social media (e.g. Facebook, Youtube)	providing information, social support
92	127	Irwin	2016	USA	RCT	social media (online program with virtual trainer and social media components)	no specific target group	physical activity	online program (with virtual trainer and social networking)	training, social support
93	128	Jaganath	2012	USA	intervention description	social media (facebook)	men who have sex with men	sexual health	social media (Facebook)	providing information, social support
94	129	Kattelmann	2014	USA	mixed methods	university	students, university (19-24)	nutrition, physical activity	online program	providing information
95	130	Kattlemann	2014	USA	RCT	university	students, university (18-24)	nutrition, physical activity	online program	providing information
96	131	Kaufmännische Krankenkasse	2020	Germany	intervention description (on website)	social media (website with social networking)	adolescents	various risk factors	website (with social networking, blog)	providing information, social support
97	132	Kim	2015	USA	RCT	social media (online program with social networking, blog)	pregnant women	nutrition, physical activity, weight (excessive gestational weight gain)	online program (with social networking, blog)	monitoring, providing information, social support
98	133	King	2016	USA	RCT	social media (app with social networking)	older adults (45+)	physical activity	smartphone (app with social networking)	monitoring, providing information, social support
99	134	Knöll	2012	Germany	thematic overview (dissertation)	community (cities), social media (social networking, virtual reality)	various target groups	various risk factors	computer program, mobile application (games with virtual reality, social networking)	environmental change, monitoring, providing information, social support
100	135	Knöll	2018	Germany	discussion paper	community	community members	health promotion in general	smartphone (app with GIS)	environmental change, partizipation, providing information, social support
101	136	Knöll	2012	Germany	intervention description	various locations (parks, streets, shopping malls, waiting halls,	various target groups	various risk factors	smartphone (app, locative health games, GPS)	environmental change, monitoring, providing information, social support
102	137	Knowlden	2015	USA	RCT	household	mothers of children (4-6)	nutrition, physical activity, weight	online program	environmental change, providing information
103	138	Knowlden	2018	USA	RCT	household	mothers of children (4-6)	physical activity, weight	online program	environmental change, providing information
104	139	Konstantinidis	2010	Greece	technology description	home	older adults	cognitive function, physical activity	computer program (game, sensors, ambient assisted living)	monitoring, training
105	140	Kousoulis	2016	Greece	mixed methods	school, social media (facebook)	students (15-18)	substance use (tobacco)	social media (facebook)	providing information, social support
106	141	Kröninger-Jungaberle	2015	Germany	mixed methods	school, university, youth group	young people (14-25)	substance use (alcohol, drugs)	videos	environmental change, providing information, social support
107	142	Kuosmanen	2018	Ireland	mixed methods	educational facility (Youthreach centres)	early school leavers (15-20)	mental health (anxiety, depression)	computer program	providing information

108	143	Kuosmanen	2018	Ireland	secondary data analysis (RCT)	educational facility (Youthreach centres)	early school leavers (15-20)	mental health (anxiety, depression)	computer program	providing information
109	144	Lakerveld	2018	Netherlands	study protocol (mixed methods)	community (supermarket)	adults (low socio economic status)	nutrition, physical activity	smartphone (app with geo-fencing)	environmental change, monitoring, providing information, social support
110	145	Lana	2013	Spain	study protocol (RCT)	school, social media (interactive website with social networking)	students (12-16), adults/teacher	various risk factors (for cancer)	website (with social networking), mobile phone (messages)	providing information, social support
111	146	Larsen	2017	USA	RCT	social media (website with social networking)	adults (Latinas)	physical activity	website (with social networking)	providing information, monitoring, social support
112	147	Laska	2016	USA	secondary data analysis (RCT)	university, social media (website with social networking)	young adults (18-35)	various risk factors (for obesity)	website (with social networking)	incentives, monitoring, providing information, social support
113	148	Lima	2018	Brazil	technology description	neighborhood	community members	transmission of tropical diseases	smartphone (app, game with virtual reality)	providing information
114	149	Little	2015	UK	RCT	household	adults	transmission of influenza	online program (with emails)	providing information
115	150	Loh	2018	USA	cross-sectional	neighborhood, social media (facebook, twitter, instagram)	parents of children (10-14)	nutrition, weight	social media (facebook, instagram, twitter), mobile phone (messages)	environmental change, incentives, providing information, social support
116	151	Lombard	2018	Australia	study protocol (mixed methods)	social media (e.g. facebook, instagram)	young adults (18-24)	nutrition	social media (e.g. facebook, instagram)	providing information, social support
117	152	Lumsden	2019	USA	pretest-posttest	dental clinic (examination room)	parents of children (2-6)	oral health	tablet application (videos)	providing information
118	153	Lyson	2019	USA	pretest-posttest	social media (platform like Twitter)	women	sexual health	social media (online platform like Twitter)	providing information
119	154	Maes	2011	Belgium	RCT	school	students (12-17)	nutrition	online program	providing information
120	155	Magoc	2015	USA	RCT	social media (online program with social networking)	students, university	physical activity	online program (eLearning with social media components)	providing information
121	156	Maher	2015	Australia	RCT	social media (facebook)	adults	physical activity	social media (facebook), pedometer	monitoring, providing information, social support
122	157	Mahmud	2013	Sweden	qualitative	social media (online program/digital health channel with social media components)	no specific target group	various risk factors	online program (digital health channel with social media components)	providing information
123	158	Malmberg	2014	Netherlands	RCT	school	students (11-15)	substance use (alcohol, tobacco, marijuana)	computer program (e-learning)	providing information
124	159	Manning	2011	USA	pretest-posttest	university, social media (facebook, MySpace)	students, university	mental health (suicide)	social media (facebook, myspace)	providing information
125	160	Manzoor	2016	Netherlands	CT	social media (online program with social networking)	no specific target group	physical activity	online program (with social networking)	monitoring, social support
126	161	Martin	2013	USA	RCT	elementary school	students (<13)	sound exposure	online program (virtual museum)	providing information
127	162	Martin	2014	USA	cross-sectional	educational facility (Head Start Centers)	mothers of children (preschool-age)	nutrition, weight	smartphone app	providing information
128	163	McCormack	2019	Canada	qualitative	community	adults	physical activity	online program (website, walkability assessment, pedometer)	environmental information, monitoring, providing information
129	164	McGloin	2015	Ireland	thematic overview (conference summary)	social media (social networking sites)	various target groups	nutrition	social media (e.g. Instagram, Facebook, Pinterest, Twitter, YouTube)	providing information, social support, monitoring, environmental change
130	165	Meng	2013	Korea	discussion paper	social media (e.g. Facebook, Twitter)	no specific target group	physical activity	social media (e.g. Facebook, Twitter)	providing information, social support
131	166	Mitchell	2019	Australia	RCT	community (rural), social media (website with social networking)	adults	physical activity	website (with social networking), mobile phone (calls)	environmental information, providing information, social support
132	167	Mitchell	2014	Australia	study protocol (RCT)	community (rural), social media (website with social networking)	adults	physical activity	website (with social networking), mobile phone (calls), pedometer	environmental information, providing information, social support
133	168	Money	2019	UK	mixed methods	household	older adults (50+)	fall prevention	computer program (game, 3D, virtual reality)	providing information
134	169	Nahm	2015	USA	secondary data analysis (RCT)	social media (online program with social networking)	older adults (50+)	bone health	online program (eLearning with social media components)	monitoring, providing information, social support, training
135	170	Neiger	2013	USA	cross-sectional	social media (twitter)	no specific target group	health promotion in general	social media (twitter)	providing information, social support
136	171	Newton	2010	Australia	RCT	school	students (13-14)	substane use (drugs)	online program	providing information
137	172	Nitsch	2019	Austria	mixed methods	school	students (14-19)	nutrition, physical activity	online program (game)	monitoring, providing information
138	173	O'Mara	2012	Australia	discussion paper	community, social media	people from diverse cultural and linguistic backgrounds (including migrants and refugees)	health promotion in general	social media (youtube, videos, digital storytelling)	providing information, social support
139	174	Paschall	2011	USA	RCT	university	students, university	substance use (alcohol)	online program	providing information
140	175	Payton	2016	USA	qualitative	social media (online platform with social networking)	students, university, female	sexual health	website (online platform with social networking)	providing information, social support
141	176	Peels	2013	Netherlands	RCT	neighborhood	older adults (50+)	physical activity	online program (with google maps)	environmental information, monitoring, providing information, social support
142	177	Peels	2012	Netherlands	mixed methods	neighborhood	older adults (50+)	physical activity	online program (with google maps)	environmental information, monitoring, providing information, social support
143	178	Peskin	2015	USA	RCT	school	students (13-14)	sexual health	online program	providing information
144	179	Phan	2016	USA	pretest-posttest	social media (online platform)	no specific target group	physical activity	website (online platform with social networking)	providing information, social support
145	180	Plaete	2016	Belgium	CT	general practice (waiting room)	patients	nutrition, physical activity	tablet application	providing information
146	181	Poppe	2018	Belgium	mixed methods	general practice (waiting room)	patients	nutrition, physical activity	tablet application	providing information
147	182	Prins	2010	Netherlands	study protocol (RCT)	school, neighborhood	students (12-13)	physical activity	computer program	environmental information, monitoring, providing information
148	183	Prins	2012	Netherlands	RCT	school, neighborhood	students (12-13)	physical activity, weight	computer program	environmental information, monitoring, providing information
149	184	Raghupathy	2012	USA	mixed methods	school	students (11-13)	substance use (drugs)	computer program	providing information
150	185	Reed-Jones	2012	USA	cross-sectional	care centers, home	older adults	fall prevention	gaming console (WiiFit)	training
151	186	Rice	2012	USA	pretest-posttest	social media (MySpace, Facebook, YouTube)	homeless young people (13-25)	sexual health	social media (MySpace, Facebook, YouTube)	providing information, social support
152	187	Rice	2011	USA	mixed methods	social media (e.g. MySpace, Facebook)	homeless young people (13-24)	substance use (alcohol, drugs)	social media (e.g. Facebook, MySpace), mobile phone	providing information, social support
153	188	Rice	2010	USA	mixed methods	social media (e.g. MySpace, Facebook)	homeless young people (13-24)	sexual health	social media (e.g. Facebook, MySpace)	providing information, social support
154	189	Rith- Najarian	2019	USA	cross-sectional	university	students, university	mental health (anxiety, depression)	online program	incentives, providing information, training
155	190	Robertson	2016	UK	secondary data analysis (RCT)	school	students (10-11)	physical activity	smartphone (app, game with GPS)	environmental information, providing information, social support
156	191	Rote	2015	USA	RCT	social media (facebook)	students, university, female	physical activity	social media (facebook), pedometer	monitoring, providing information, social support
157	192	Rovniak	2013	USA	study protocol (RCT)	social media (online program with social networking)	adults (35-64)	physical activity	online program (with social networking, GPS)	environmental information, monitoring, providing information, social support
158	193	Schoene	2013	Australia	RCT	independent-living units of a retirement village, home	older adults (65+)	fall prevention	gaming tool (computerized step pad, TV)	training
159	194	Schwinn	2014	USA	RCT	public housing	adolescents, female (10-12)	nutrition, physical activity, substance use (drugs)	online program	providing information
160	195	Shafii	2019	USA	RCT	sexually transmitted infection clinic (waiting or exam room)	adolescents (14-24)	sexual health	computer program	providing information
161	196	Shanta Bridges	2018	USA	pretest-posttest	university	students, university	mental health (depression, suicide)	online program	providing information

162	197	Sharma	2015	USA	RCT	school	students (9-11)	nutrition, physical activity	computer program (game)	providing information
163	198	Shegog	2017	USA	mixed methods	school	students (9-16)	sexual health	online program	providing information
164	199	Smith	2014	USA	discussion paper	social media (e.g. Facebook, Twitter)	people from the rural USA/Mexico border region	health promotion in general	social media (e.g. Twitter, Facebook)	providing information
165	200	West	2016	USA	RCT	university, social media (facebook)	students, university	physical activity, weight	social media (facebook)	environmental information, monitoring, providing information, social support
166	201	Springvloet	2014	Netherlands	study protocol (RCT)	household	adults	nutrition	online program	environmental change, providing information
167	202	Suomi	2014	Finland	cross-sectional	virtual world (online program)	no specific target group	various risk factors	online program (with virtual world)	empowerment, environmental change, providing information, social support
168	203	Tague	2014	Australia	cross-sectional	social media (website with social networking)	no specific target group	physical activity	website (with social networking)	providing information, social support
169	204	Techniker Krankenkasse	2019	Germany	intervention description (on website)	university	students, university	physical activity	videos	providing information, training
170	205	Teitelman	2018	USA	cross-sectional	social media (app with social networking)	women, young (18-26)	vaccination	smartphone (app with social networking)	providing information, social support
171	206	Tolks	2019	Germany	qualitative	social media (online program with social networking)	no specific target group	wellbeing in general	online program (game with social networking)	monitoring, social support
172	207	Tortolero	2010	USA	RCT	school	students (12-13)	sexual health	online program	providing information
173	208	Tsai	2013	Taiwan	mixed methods	assisted living community	older adults (60+)	physical activity	computer program (game, 3D environment, virtual reality, with sensors)	training
174	209	van Kessel	2016	Australia	qualitative	social media (e.g. facebook, instagram)	adolescents, female (13-18)	physical activity	social media (e.g. facebook, instagram)	providing information, social support
175	210	van Lippevelde	2016	Belgium	CT	school, social media (smartphone app with social networking)	students (14-16)	nutrition	smartphone app (game with virtual environment and social networking)	incentives, monitoring, providing information, social support
176	211	Van Stralen	2010	Netherlands	RCT	neighborhood, social media (website with social networking)	older adults (50+)	physical activity	website (with social networking, GIS)	environmental information, providing information, social support
177	212	Van Stralen	2011	Netherlands	RCT	neighborhood, social media (website with social networking)	older adults (50+)	physical activity	website (with social networking, GIS)	environmental information, providing information, social support
178	213	Walton	2014	USA	RCT	primary care clinic	adolescents (12-18)	substance use (cannabis)	computer program	providing information
179	214	Ward	2017	USA	RCT	child care centers	child care center directors who care for children (3-5)	nutrition	computer program	environmental change
180	215	Washington	2015	USA	pretest-posttest	church	church members	physical activity	online program	providing information
181	216	Watkins	2019	USA	study protocol (mixed methods)	social media (facebook)	men, young black	mental health (depression)	social media (facebook)	providing information, social support
182	217	Watkins	2016	USA	secondary data analysis (mixed methods)	social media (facebook)	men, young black (18-26)	mental health (depression)	social media (facebook)	providing information, social support
183	218	Webster	2015	UK	qualitative	sexual health clinics (waiting room)	men	sexual health	website (on tablet)	providing information
184	219	Whittmore	2013	USA	mixed methods	school	students (12-19)	nutrition, physical activity, weight	online program	providing information
185	220	Widman	2017	USA	secondary data analysis (RCT)	school	students, female (<18)	sexual health	online program	providing information
186	221	Widman	2019	USA	RCT	school	students (15-18)	sexual health	online program	providing information
187	222	Widman	2018	USA	RCT	school	students, female (14-15)	sexual health	online program	providing information
188	223	Winskell	2019	USA	mixed methods	social media (smartphone app with social networking)	adolescents (11-14)	sexual health	smartphone (app, interactive game)	providing information, social support
189	224	Witzel	2016	UK	qualitative	social media (facebook, twitter)	men (gay and bisexual), african people	sexual health	social media (facebook, twitter)	providing information, social support
190	225	Wright	2019	Australia	qualitative	social media (social networking sites)	no specific target group	health promotion in general	social media (social networking sites)	environmental change, monitoring, providing information, social support
191	226	Ybarra	2012	Uganda	mixed methods	school	students (13-18)	sexual health	online program	providing information
192	227	Yepes	2015	Switzerland	RCT (posttest-only)	restaurant	young adults (18-33)	nutrition	tablet application	providing information
193	228	Yoost	2017	USA	pretest-posttest	school	students, female (14-18)	sexual health	computer program (teleconference)	providing information
194	229	Youm	2015	Korea	cross-sectional	neighborhood, social media (u-healthcare center with social networking)	patients	various risk factors	u-healthcare center (with social networking)	monitoring, providing information, social support
195	230	Young	2013	USA	RCT	social media (facebook)	men who have sex with men	sexual health	social media (facebook)	providing information, social support
196	231	Young	2014	USA	secondary data analysis (RCT)	social media (facebook)	men who have sex with men	sexual health	social media (facebook)	providing information, social support
197	232	Young	2013	USA	pretest-posttest	social media (facebook)	men who have sex with men	sexual health	social media (facebook)	providing information, social support
198	233	Zhang	2015	USA	RCT	social media (website with social networking)	students, university	physical activity	website (with social networking)	providing information, social support
199	234	Zhang	2016	USA	RCT	social media (website with social networking)	students, university	physical activity	website (with social networking)	incentives, providing information, social support
200	235	Zhao	2016	USA	cross-sectional	household	no specific target group	physical activity	computer program (Microsoft Kinect), sensors (smartwatch)	monitoring
