# Supplementary figures and images for: Digital Health Promotion and Prevention in Settings: Scoping Review
Source: J Med Internet Res. 2022 Jan 28;24(1):e21063. doi: 10.2196/21063 (PMC8838600; doi:10.2196/21063)

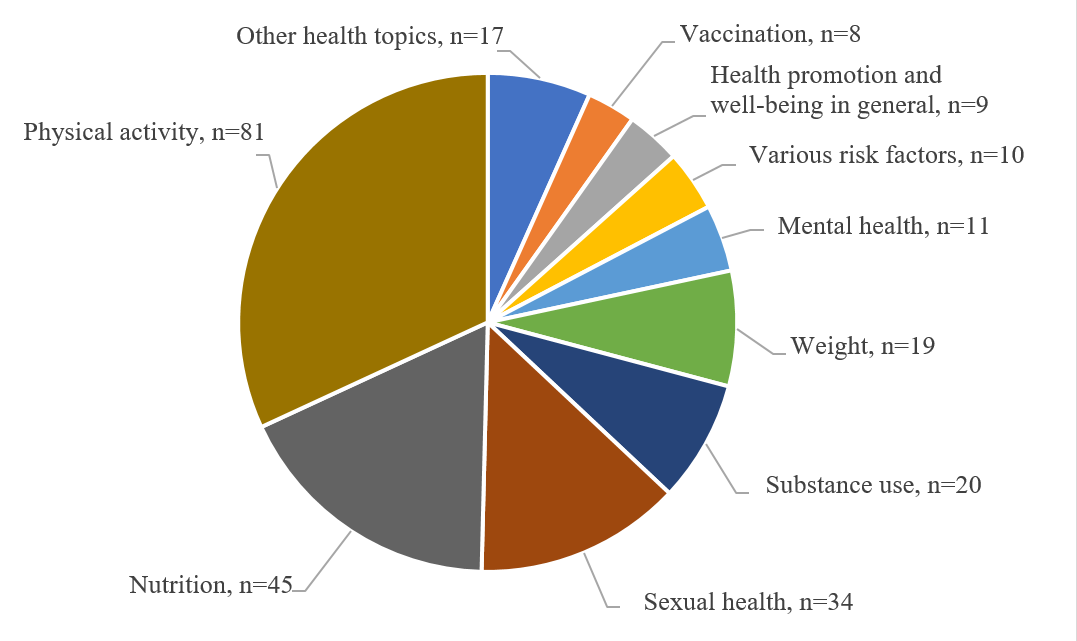

Supplement: Multimedia Appendix 3 [file jmir_v24i1e21063_app3.png]
